# COMMUNITY PERSPECTIVES ON COMMUNICATION DISPARITIES IN LATE OLD AGE AND TACTICS TO CONNECT WITH THE HARDLY REACHED

**DOI:** 10.1093/geroni/igac059.1450

**Published:** 2022-12-20

**Authors:** Carrie Leach, Thomas Jankowski

**Affiliations:** Wayne State University, Detroit, Michigan, United States; Wayne State University, Detroit, Michigan, United States

## Abstract

Individuals who face the greatest health burdens often have the fewest communication resources to draw from to help them cope. Communication infrastructure theory provides a framework for understanding how communication resources and context constrain and enable connections to health enhancing information. Data collected from 1,609 older adults through in-depth interviews and a random sample survey in one midwestern county in Michigan as part of a multi-year, multi-level community engaged study and a statewide random digit dial telephone survey showed that age influenced integration in a communicative structure. Findings suggest that the dynamics of living into late old age result in communication disparities through diminishing interpersonal resources, activity, and opportunities to congregate. Actionable knowledge and strategies provided to community leaders on how to connect with the hardly reached, individuals age of 75 and older, are presented.

